# Laboratory spectral X-ray tomography for the differentiation of common rock-forming minerals

**DOI:** 10.1038/s41598-026-47955-z

**Published:** 2026-04-30

**Authors:** K. Kularatne, O. Darouich, P. Sénéchal, P. Moonen, H. Derluyn

**Affiliations:** 1 Universite de Pau et des Pays de l’Adour, CNRS, LFCR, Pau, France; 2 Universite de Pau et des Pays de l’Adour, CNRS, DMEX, Pau, France; 3 Present Address: LCMCP (UMR 7574), Paris, France

**Keywords:** X-ray spectral tomography, X-ray micro-tomography, Linear attenuation coefficient, Multi-energy histogram, Segmentation, Rock-forming minerals, Mineralogy, Petrology, Imaging techniques

## Abstract

Conventional X-ray micro computed tomography (micro-CT) is widely used for non-invasive 3D material characterization, yet differentiating between the material constituents can be challenging. This is notably the case when characterizing rocks from the Earth’s crust. About 95% of them is composed of a mixture of common rock-forming minerals, many of which are indistinguishable with conventional micro-CT. We overcome this limitation by combining laboratory spectral micro computed tomography (sp-CT) with an astute analysis workflow. Where previous studies showed laboratory sp-CT’s potential for identifying heavy elements, we focus on the light-element-bearing minerals that do not feature characteristic absorption edges within the detectable energy range of state-of-the art spectral detectors. For a natural monzo-diorite sample, we show that those minerals appear as separate clusters on a multi-energy histogram derived from a laboratory sp-CT tomogram. Selecting a particular cluster is equivalent to segmenting the corresponding constituent. The segmented phases were corroborated by scanning electron microscopy and Raman spectroscopy. The presented workflow is applicable to any multi-component material. Specifically, for the Earth Sciences, it breaks the barrier to analyse a variety of silicate-bearing rocks, their minerals, structures and reactions in three dimensions, non-invasively in the laboratory.

## Introduction

X-ray micro computed tomography (micro-CT) is a key technique for non-destructive 3D imaging of materials in various research fields^1,2^. In geosciences, micro-CT is widely used for 3D analysis of porous rock^3^, mineral and salt crystallization^4–6^, fossils^7,8^, fractures^9,10^, ores^11^, rock deformation^12^ and for in-situ monitoring of dynamic processes^13–15^. Image formation in conventional micro-CT is based on the X-ray attenuating properties of materials, that are dependent on both their density and elemental composition^10^. The measurement yields a 3D image composed of voxels, where each voxel is characterized by a linear attenuation coefficient. Distinct values for the linear attenuation coefficient correspond to different material phases, allowing phases with contrasting attenuation to be easily identified on the micro-CT image. However, many combinations of composition and density yield a similar linear attenuation coefficient, and distinguishing them by means of conventional micro-CT is therefore challenging. This is the case for most rock forming minerals.

The majority of rocks in the Earth’s crust are composed of common minerals such as quartz (SiO_2_), orthoclase feldspar (KAlSi_3_O_8_), plagioclase feldspar (NaAlSi_3_O_8_ and CaAl_2_Si_2_O_8_), micas (e.g. biotite, K(Mg, Fe)_3_(AlSi_3_O_10_)(F, OH)_2_), olivines ((Mg, Fe)_2_SiO_4_), pyroxenes (e.g. enstatite, Mg_2_SiO_4_) and amphiboles (e.g. hornblende, (Ca, Na, K)_2_(Mg, Fe^2+^,Fe^3+^,Al)_5_[Si_6_(Al, Si)_2_O_22_](OH, F)_2_)^16,17^. In their pure form, these minerals consist of H, O, F, Na, Mg, Al, Si, K, Ca, and Fe combined in different atomic proportions. Therefore, these minerals can be distinguished by using 2D imaging techniques such as scanning electron microscopy or transmission electron microscopy with energy dispersive spectroscopy (SEM EDS, TEM EDS), or electron microprobe analysis (EMPA), that are all based on the elemental composition. To get 3D elemental maps, one could use a focused ion beam (FIB) to liberate a surface of interest, and apply SEM EDS on that surface. The drawback is that this technique is destructive. Micro-CT can be used to non-invasively inspect large sample volumes, yet, as the densities of above minerals differ only slightly (2.6 to 3.0 g/cm^3^)^16^ and their prime constituents cover only a limited atomic range (from Z = 8 for Oxygen up to Z = 26 for Iron), the corresponding linear attenuation coefficients for X-rays are close, resulting in poor contrast. In order to differentiate between those rock forming minerals in 3D X-ray images, different contrast mechanisms must be exploited. Techniques such as dual energy imaging^18–23^ phase contrast imaging^24–26^ or correlative imaging^12,27–30^ are actively explored to solve this challenge. The recent introduction of spectral detectors into laboratory CT scanners constitutes an alternative path^31,32^.

Laboratory spectral CT (sp-CT) shares many characteristics with conventional micro-CT. Both methods rely on an X-ray source that produces a polychromatic X-ray beam with a given spectrum. That -often conical- beam subsequently travels through the material of interest and impinges on a detector. In the case of conventional micro-CT, the total intensity of the incident beam is recorded, whereas in spectral CT the energy of each incident photon is detected separately. A brief introduction on the technology is given in the review by Ballabriga et al.^33^. The measured energies of all photons are collected in energy bins and approximate the incident spectrum, the integral of which is the signal detected in conventional micro-CT. In a given energy bin, the relation between the intensity before, $\:{I}_{0}$, and after passage, $\:I$, through a material of thickness $\:d$ is expressed by the well-known Beer-Lambert law:

$$\:I\left(E\right)={I}_{0}\left(E\right)\mathrm{exp}\left[-\mu\:\left(E\right)d\right]$$

In this expression, $\:\mu\:\left(E\right)={\mu\:}_{m}\left(E\right)\rho\:$ is the linear attenuation coefficient of the material, which corresponds to the product of the material-specific and energy-dependent mass attenuation coefficient $\:{\mu\:}_{m}\left(E\right)$ and the material density $\:\rho\:$. The attenuated energy spectrum $\:I\left(E\right)$ is recorded in each detector pixel and this for different orientation angles of the sample. If the energy bin size is sufficiently small to be considered monochromatic, an inverse Radon transform yields $\:\stackrel{-}{\mu\:}\left(E\right)=\stackrel{-}{{\mu\:}_{m}}\left(E\right)\stackrel{-}{\rho\:}$ for each voxel (i.e., a pixel in three dimensions) of the 3D output volume. Hereby $\:\stackrel{-}{{\mu\:}_{m}}\left(E\right)$ is responsible for the energy-dependency of the reconstructed signal and $\:\stackrel{-}{\rho\:}$ acts as a scaling factor. The overbar notation ($\:\stackrel{-}{\cdot\:}$) indicates that the reconstructed signal approximates the true physical quantity represented by the dot (•).

Theoretical calculations of the energy-dependent mass attenuation coefficient $\:{\mu\:}_{m}\left(E\right)$ are available for most elemental media^34^. When this quantity exhibits detectable characteristic features, a direct comparison between the shape of the reconstructed and theoretical signal is straightforward, enabling voxel-level material identification. Absorption edges, i.e., sudden changes in mass attenuation, are a typical example of such characteristic features. Sittner et al.^35^ exploited the absorption edges obtained with laboratory spectral CT to identify heavy elements such as W (Z = 74), Au (Z = 79) and Pb (Z = 82). In addition, sp-CT has been used to identify heavy-element-bearing minerals (Barite, Ceria, Galena, Parisite, Electrum, Monazite, Cassiterite, etc.) inside materials^36,37^, an approach similar to that used for SEM-based automated mineralogical classification^38^. In many cases however, such as the case of common rock forming minerals, the absorption edges of the elements of interest fall below the practical energy range of most spectral detectors (Hexitec^39^, Medipix4/timepix4^40^, Eiger2^41^ and Pilatus4^42^ all have a low energy threshold around 4-5 keV, i.e. excluding absorption edge detection of elements with Z < 20).

In the current report, we propose a strategy to differentiate between phases with similar linear attenuation coefficients by performing laboratory sp-CT imaging in combination with a tailored image postprocessing workflow, inspired by dual-energy CT. Herein, two energy intervals from the full detected range are considered. If $\:{{\mu\:}_{m}}_{\left\{{E}_{1}\right\}}$ and $\:{{\mu\:}_{m}}_{\left\{{E}_{2}\right\}}$ represent the mass attenuation coefficient averaged over energy ranges $\:{E}_{1}$ and $\:{E}_{2}$, respectively, one can plot $\:{{\mu\:}_{m}}_{\left\{{E}_{1}\right\}}\rho\:$ against $\:{{\mu\:}_{m}}_{\left\{{E}_{2}\right\}}\rho\:$ for each voxel in the dataset. All voxels with the same chemical composition will cluster around a line in this representation, whereby the slope $\:{{\mu\:}_{m}}_{\left\{{E}_{1}\right\}}/{{\mu\:}_{m}}_{\left\{{E}_{2}\right\}}$ is composition dependent and the distance to the origin is solely a function of density. In contrast to dual energy CT, the energy ranges $\:{E}_{1}$ and $\:{E}_{2}$ are selected a posteriori, i.e., after data acquisition, and can be optimized for each material phase separately to maximize the differentiating power from the other material phases. We develop our strategy using a natural monzo-diorite sample composed primarily of common rock-forming silicate minerals. The workflow is further validated by means of independent complementary analytical techniques applied on the same sample. The resulting methodology is broadly applicable to any material composed of multiple phases, as recently demonstrated by the successful differentiation between carbonate minerals, such as calcite, magnesian calcite, dolomite and magnesite^43^.

## Results

**Fig. 1:**
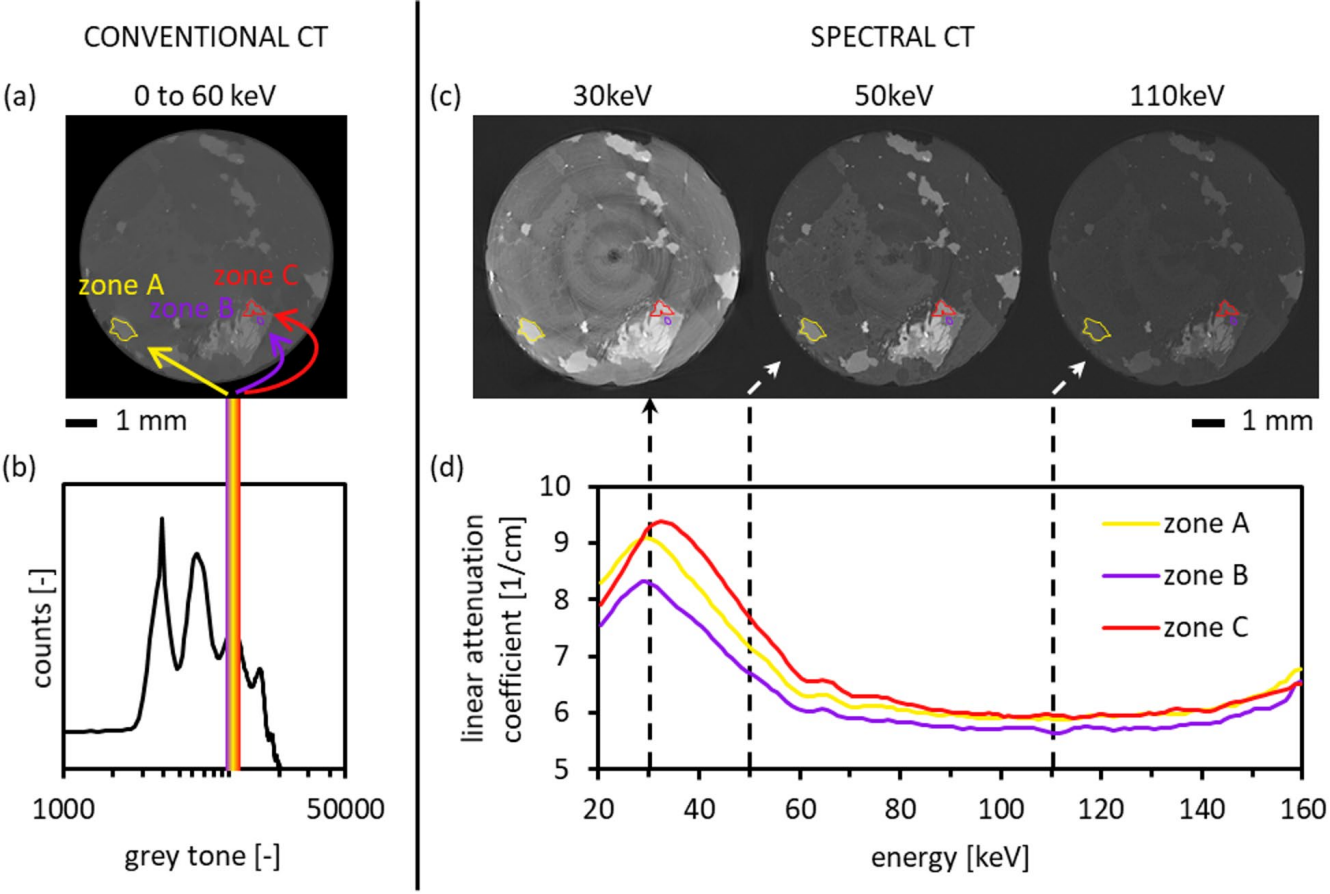
The same cross-sectional slice (**a**) imaged with conventional micro-CT imaging and (**b**) its corresponding grey tone histogram, and (**c**) imaged with spectral CT imaging at 30 keV, 50 keV and 110 keV. (**d**) Energy-dependent linear attenuation coefficient corresponding to zones A, B and C.

We develop our methodology using a cylindrical natural monzo-diorite sample (8.75 mm in diameter) from Kansas, USA, containing light-element-bearing minerals. Its circular, polished upper surface is representative for the mineral composition of this rock. Figure 1a shows a transverse slice through the reconstructed 3D dataset acquired with conventional micro-CT imaging. The selected slice is located immediately below the polished surface that will be analysed by complementary characterization techniques to validate our methodology. Figure 1b depicts the corresponding grey-tone histogram of the raw data, which reflects the variation in linear attenuation coefficients in the sample. For example, the zones A, B and C, contoured on Fig. 1a, present a similar grey tone range, as shown on Fig. 1b. It is clear that conventional X-ray imaging will not enable to accurately differentiate between those phases based on contrast in grey tone. Sp-CT yields an energy-dependent signal at each voxel, as opposed to a single value for conventional micro-CT. Sp-CT images corresponding to three arbitrarily selected energies are presented on Fig. 1c. The grey tone of these images is proportional to the linear attenuation coefficient, where darker tones correspond to lower attenuation. Figure 1d presents the variation of the measured linear attenuation coefficient with energy for zones A, B and C over the entire spectral detector energy range. The three curves feature different peak positions and are non-overlapping in the range below 70 keV (Fig. 1d). Sp-CT will thus allow for their differentiation. Note that the (raw) measured linear attenuation coefficients need to be corrected if one aims to recover the true coefficients, as e.g. tabulated by NIST. This is not required for material differentiation.

**Fig. 2:**
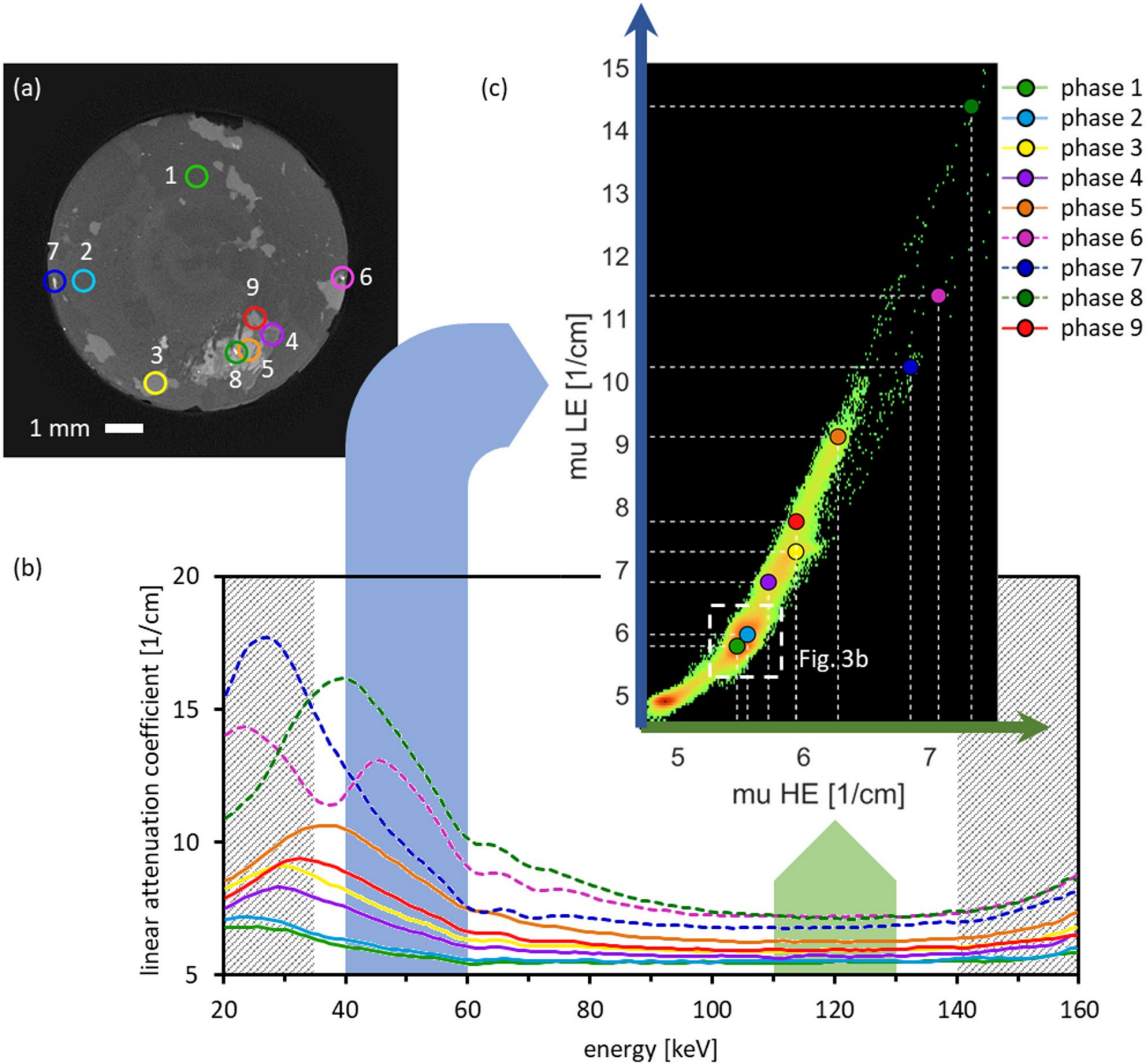
(**a**) Sp-CT image at 50 keV. Circles #1 to #9 mark 9 different phases present in the slice. Circles are scaled with a factor 10 for better visibility. (**b**) Zone-averaged energy-dependent linear attenuation coefficient of these zones between 20 and 160 keV. (**c**) Multi-energy histogram of the sp-CT image obtained for the low energy (LE) range 40–60 keV versus the high energy (HE) range 110–130 keV. The centre of the circular markers superimposed on the histogram highlights the position where each zone depicted on (**a**) can be found.

The applied procedure starts by visually exploring various zones on the sp-CT dataset and retaining those corresponding to phases with dissimilar linear attenuation coefficients. In this study, nine phases numbered from 1 to 9 were distinguished. The locations where they were encountered are indicated on Fig. 2a and the measured linear attenuation coefficients at those locations are presented on Fig. 2b. Note the small but spurious wrinkles in all curves around 60 and 70 keV, originating from the K-α and K-β fluorescence peaks of the tungsten target of the X-ray tube, and the small trend upward towards higher energies, corresponding to charge pile-up. Most curves (solid lines) do not exhibit distinct characteristic features (absorption edges), rendering direct identification of elements difficult. The three dashed lines feature something that could be an absorption edge, yet for two of them the feature lies in a region that is potentially less reliable. Only for the purple line (phase 6), the absorption edge is reliable.

After this preliminary analysis, the dual-energy inspired approach is used to separate each phase. We start by defining a low (here 40-60 keV) and a high energy range (here 110-130 keV). In the low-energy range, attenuation is typically governed by photo-electric absorption, while Compton scattering dominates in the high-energy range. Both attenuation mechanisms depend on the density and the atomic number of the traversed material phase, but in a different way, hence providing complementary information that will be exploited for phase differentiation. For each voxel of the spectral dataset, we calculate the average linear attenuation coefficient over both energy ranges, and these uniquely define the location of that voxel in a multi-energy histogram (Fig. 2c). Hotter colours correspond to regions within the histogram containing a higher voxel density. The data clearly cluster around multiple lines. Upon more detailed inspection, different clusters (i.e. regions with a higher voxel density) can be observed, each corresponding to a material phase. The bright red spot around (5,5) in Fig. 2c corresponds for example to the air around the sample.

**Fig. 3:**
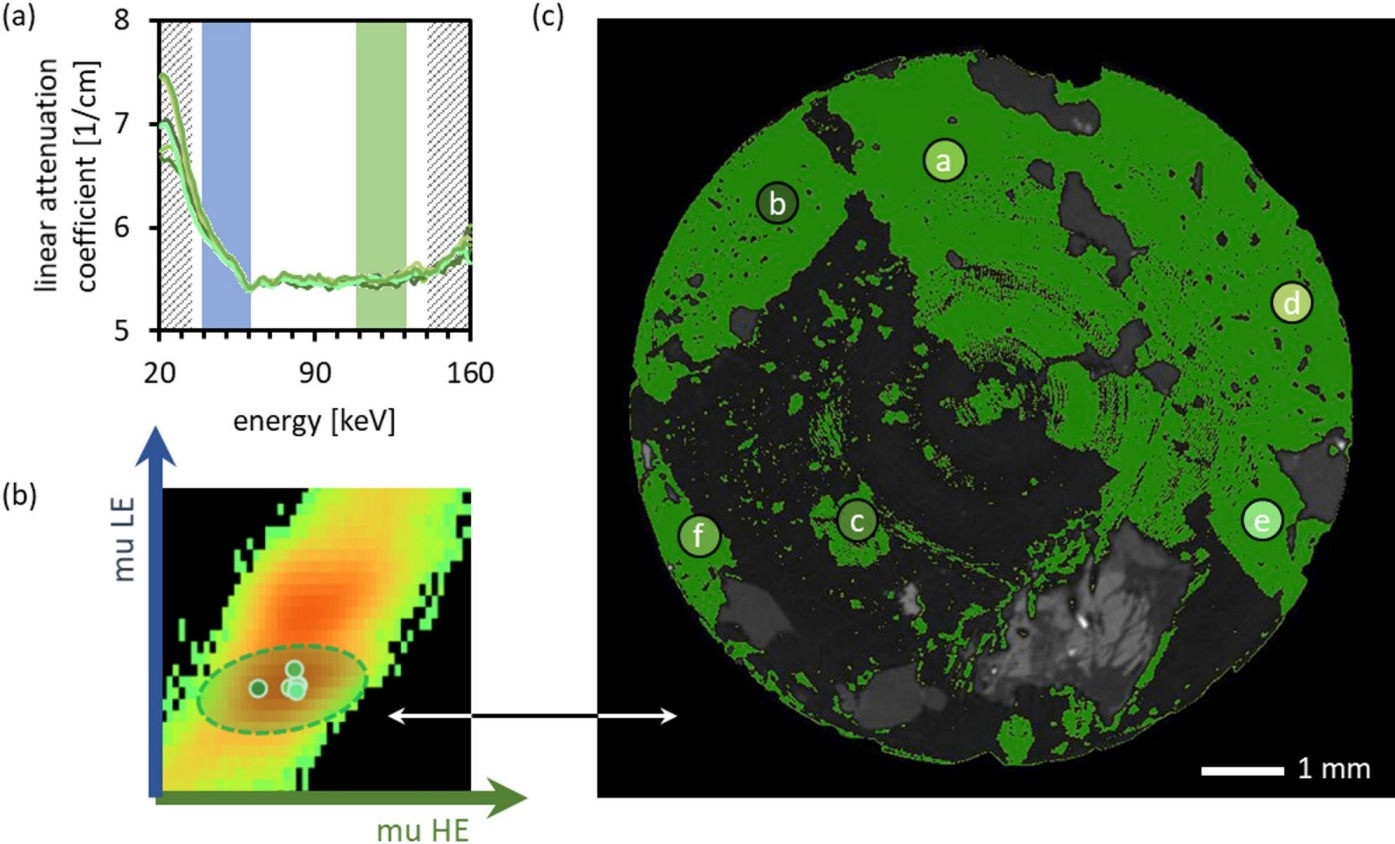
Example of segmentation of phase#1: (**a**) energy-dependent linear attenuation coefficient of the areas #a to #f selected on (**c**), the sp-CT image at 50 keV. (**b**) Zoom on the multi-energy histogram at the location indicated in Fig. 2c. The six areas (#a to #f) are indicated by the six circular markers. The cluster comprising these areas covers phase #1, segmented in green on (**c**).

Segmentation of a cluster in the multi-energy histogram corresponds to segmenting a group of voxels with a similar composition and density in the sp-CT dataset. For example, the cluster within the dashed ellipse on the zoom of the multi-energy histogram (Fig. 3b) corresponds to the green area shown on Fig. 3c. The energy-dependent linear attenuation coefficients of six arbitrarily selected voxels belonging to this cluster are depicted on Fig. 3a and their similar shape between 35 keV and 140 keV is a strong indication that they have a similar chemical composition. Below 35 keV and above 140 keV, the obtained linear attenuation coefficients deviate because of the low transmission for low-energy photons and pile-up effects, respectively. These regions, hatched in Fig. 3a, are excluded from the analysis.

**Fig. 4:**
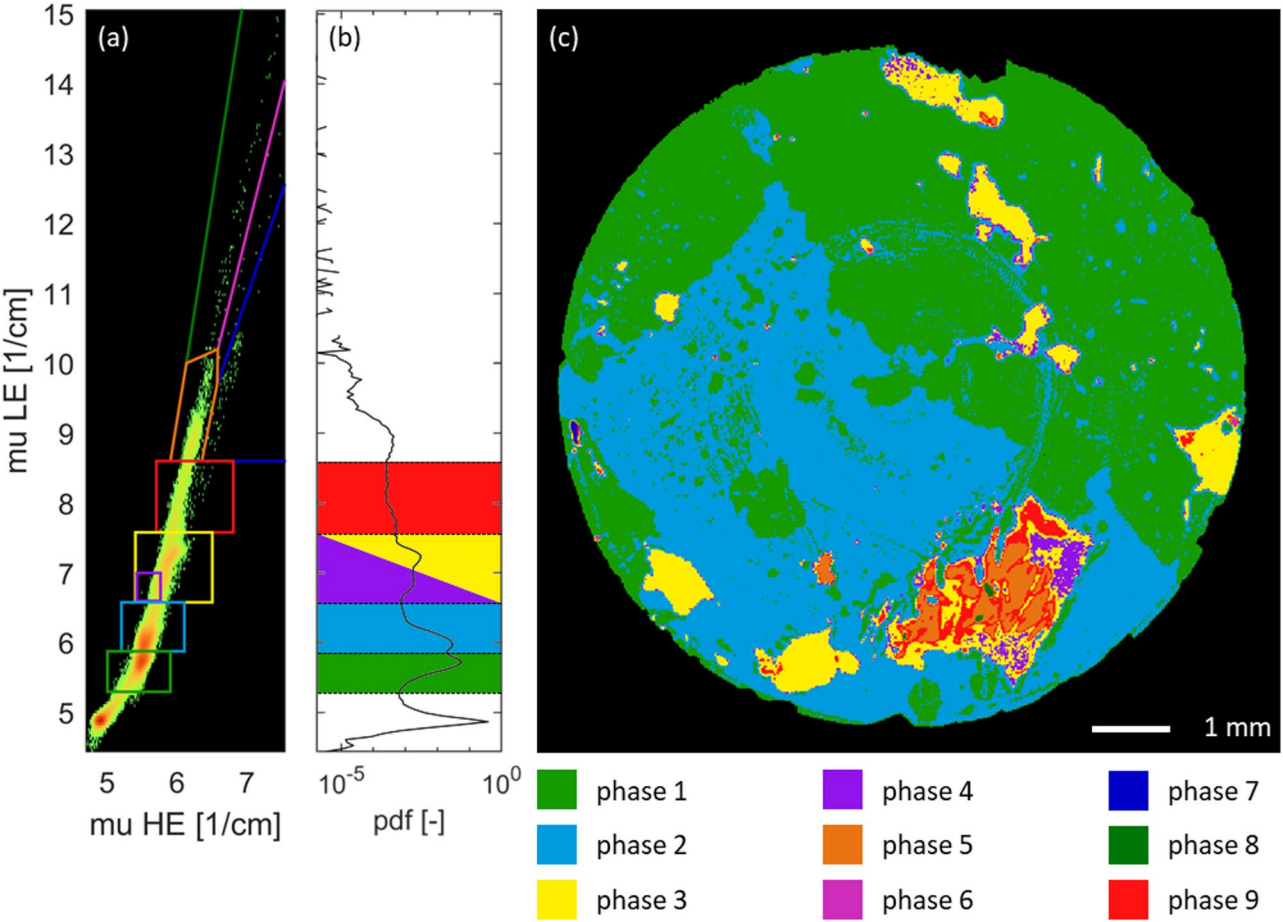
(**a**) Segmentation of the multi-energy histogram, partly based on the horizontally-projected histogram depicted in (**b**) and resulting in the segmented image shown in (**c**).

By segmenting the multi-energy histogram, it is thus possible to segment phases in the sp-CT dataset. In the case at hand, two complementary segmentation strategies were applied. For the lowest attenuating elements, the thresholds were selected based on the one-dimensional histogram obtained by projecting the multi-energy histogram along the horizontal direction (Fig. 4b). Each relative maximum is considered to be the centre of a cluster, and each relative minimum was taken as a threshold separating the neighbouring phases. The width of the boxes on Fig. 4a is taken as such as to cover all data points. As only a limited volume fraction of the material was composed of more attenuating material phases, the point density in the multi-energy histogram is low for high µ, and hence the statistics are too poor to reliably segment based on the projected histogram. Therefore, a different strategy is applied. Each point in the multi-energy histogram is given by the following coordinate: $\:\left({{\mu\:}_{m}}_{\left\{{E}_{2}\right\}}\rho\:,{{\mu\:}_{m}}_{\left\{{E}_{1}\right\}}\rho\:\right)$. It is clear that the density $\:\rho\:$ acts as a scaling factor, while the ratio $\:{{\mu\:}_{m}}_{\left\{{E}_{1}\right\}}\rho\:/{{\mu\:}_{m}}_{\left\{{E}_{2}\right\}}\rho\:$ represents a slope. Therefore, points that cluster around a line have a similar material composition ($\:\mu\:$), but a different density ($\:\rho\:$). This justifies segmenting phases 6, 7 and 8 based on the slope of their point distribution in the multi-energy-histogram. Additionally, it was verified that the full spectra of phase 6 feature a K-edge (as observed on Fig. 2). The resulting segmentation of the multi-energy histogram is depicted on Fig. 4a, and the corresponding segmented slice is shown in Fig. 4c. For completeness, we mention that the exact position of the boundary separating phases 3 and 4, and between phases 5 and 8, in the multi-energy histogram is prone to some degree of uncertainty. The effect of moving this boundary is that the physical interface between these phases displaces. Here, the boundaries were positioned by trial and error so that the resulting segmentation was visually overlapping with the features visible on the images.

The described workflow is performed using an in-house software tool for post-processing sp-CT data. The tool covers the described steps, ranging from extracting attenuation profiles, over selecting adapted energy ranges and constructing the multi-energy histogram, up to segmenting the latter and inspecting the corresponding result. The code is available on Git alongside this report (https://git.univ-pau.fr/dmex/segmentSpectralData/).

**Fig. 5:**
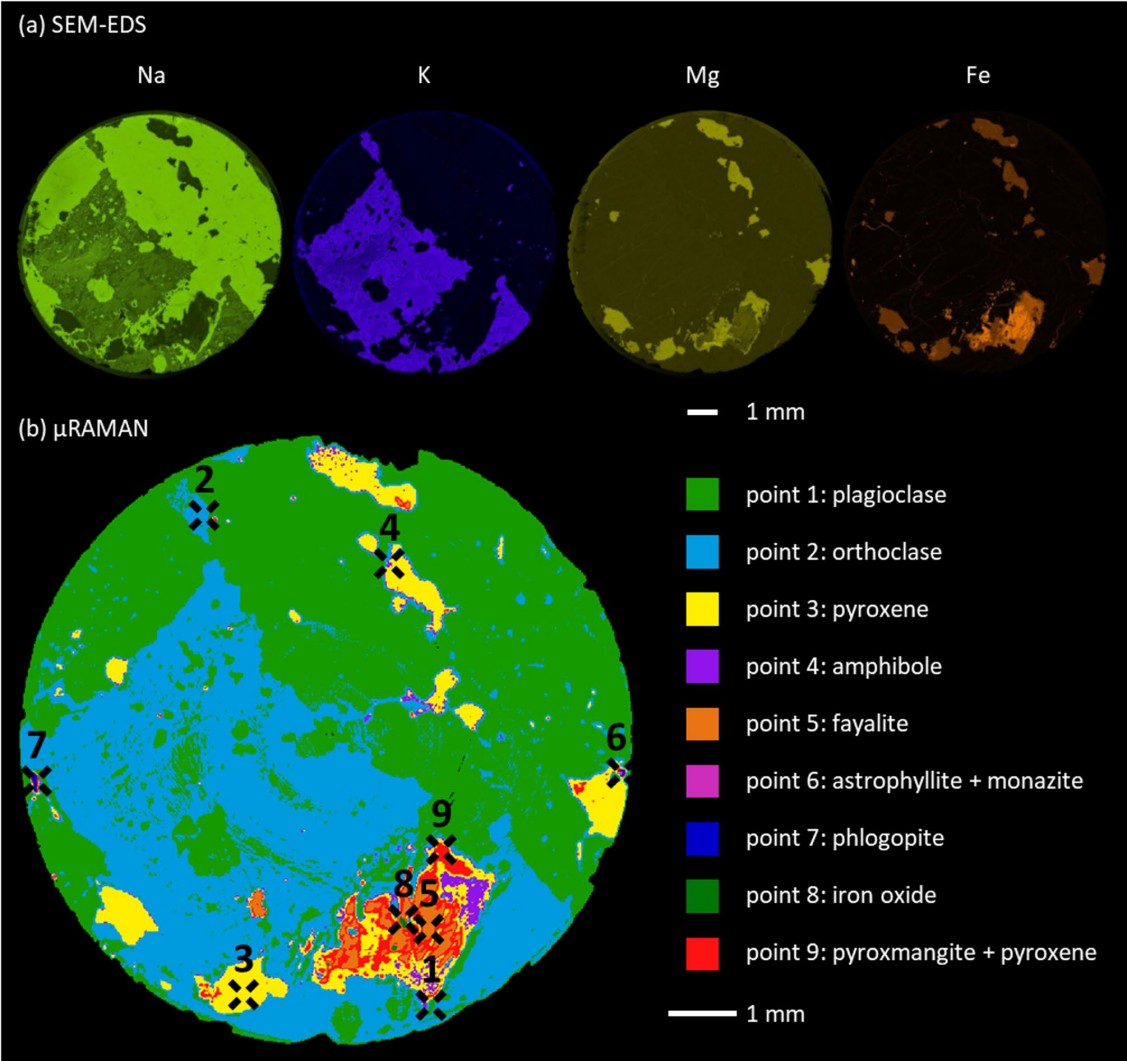
(**a**) SEM-EDS maps of Na, K, Mg and Fe, supporting the identification of phases 1–5; (**b**) segmented sp-CT image with indication of 9 points (one per phase) at which µRaman was performed and the independently identified minerals.

The segmentation obtained with the sp-CT method was corroborated using two complementary methods, 2D SEM EDS element distribution mapping and µRaman spectroscopy. Table 1 summarizes the identified phases, their chemical compositions, and the method/s used for their identification. Figure 5 summarizes the key-results from both techniques.

The segmentation of phase 1 matches well with the SEM EDS Na distribution map (Fig. 5a). µRaman spectroscopy identifies this phase as plagioclase, one of the most Na-rich phases in the sample (Fig. 5b). The segmentation of phase 2 corresponds well with the SEM EDS K distribution map, identified with µRaman spectroscopy as orthoclase, one of the most K-rich phases in the sample. Phases 3 and 4 match with the combination of SEM EDS Mg and Fe distribution maps (Fig. 5a). µRaman spectroscopy identifies those phases as pyroxene and amphibole, respectively. Phase 5 corresponds to highly Fe-rich zones in the SEM EDS Fe distribution map (Fig. 5a), identified by µRaman spectroscopy as fayalite. Phases 6–9 represent a small volume fraction of the analysed section. µ-Raman spectroscopy confirmed the existence of these phases identified with sp-CT and enabled identifying the corresponding minerals.

**Table 1:** Identification of phases present in the natural monzo-diorite sample from Kansas, USA.

Phase#	Corresponding mineral	Chemical composition	Technique/s used to identify the mineral
Phase 1	Plagioclase	Na_0.8_ Al_1.2_Ca_0.2_Si_2.8_O_8_^*^	SEM EDS, µRaman
Phase 2	Orthoclase	K_0.8_Na_0.2_AlSi_3_O_8_^*^	SEM EDS, µRaman
Phase 3	Pyroxene	Fe_1.6_Mg_0.5_Mn_0.1_Si_2_O_6_^*^ or FeCa_0.7_Mg_0.3_Si_2_O_6_	SEM EDS, µRaman
Phase 4	Amphibole	Fe_3.5_Al_1.9_Ca_1.7_Mg_1.1_Na_0.4_K_0.2_Si_6_O_22_(OH)_2_^*^	SEM EDS, µRaman
Phase 5	Fayalite	Fe_1.8_Mn_0.1_Mg_0.1_SiO_4_^*^	SEM EDS, µRaman
Phase 6	Astrophyllite + monazite	(K, Na)_3_(Fe^2+^,Mn)_7_Ti_2_Si_8_O_24_(O, OH)_7_^$^ + (Ce, La, Th)PO_4_^$^	µRaman
Phase 7	Phlogopite	KMg₃AlSi₃O₁₀(F, OH)₂ ^$^	µRaman
Phase 8	Iron-oxide	Fe_2_O_3_^$^	µRaman
Phase 9	Pyroxmangite + pyroxene	MnSiO_3_^$^ + Fe_1.6_Mg_0.5_Mn_0.1_Si_2_O_6_^*^ or FeCa_0.7_Mg_0.3_Si_2_O_6_	µRaman

*Composition obtained by SEM-EDS^44^.

^$^Generalized chemical formulae of mineral^16,17^.

**Fig. 6:**
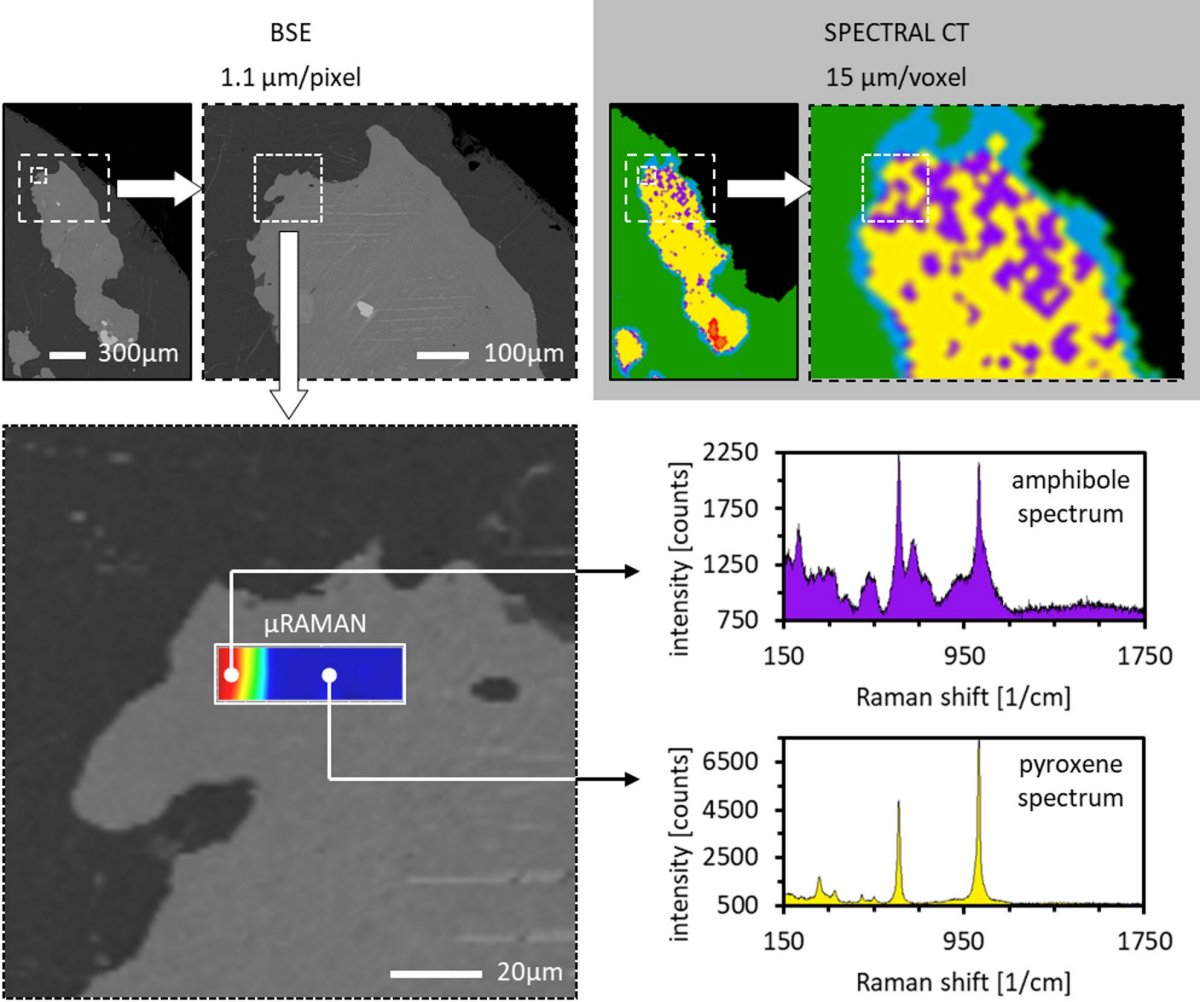
Distinguishing pyroxene and amphibole: SEM BSE image and segmented spectral CT image of a zone containing both minerals. µRaman mapping and corresponding µRaman spectra confirm the presence of amphibole (purple spectrum and purple segments in the sp-CT image) and pyroxene (yellow spectrum and yellow segments in the sp-CT image).

We further focus on the distinction between pyroxene (phase 3) and amphibole (phase 4). In this natural monzo-diorite sample, pyroxene has been partially transformed into amphibole. This reaction has been observed on SEM BSE images and the two minerals were retrieved by the sp-CT segmentation (Fig. 6). µRaman mapping was conducted to trace the reaction front and to confirm the two minerals. The red area on the µRaman map indicates the zone containing amphibole and the blue area indicates the zone containing pyroxene. The corresponding µRaman spectra with characteristic peaks of amphibole and pyroxene are given in Fig. 6 as well. Note that the BSE images and µRaman spectra reported in Fig. 6 were obtained on the opposite surface from the one analysed with spectral CT after cutting the sample, explaining the small differences in the shape of all interfaces. Note also the difference in resolution between the BSE dataset (1.1 μm/pixel) and the sp-CT dataset (15 μm/voxel), explaining why the phase boundaries are better defined in the BSE dataset.

**Fig. 7:**
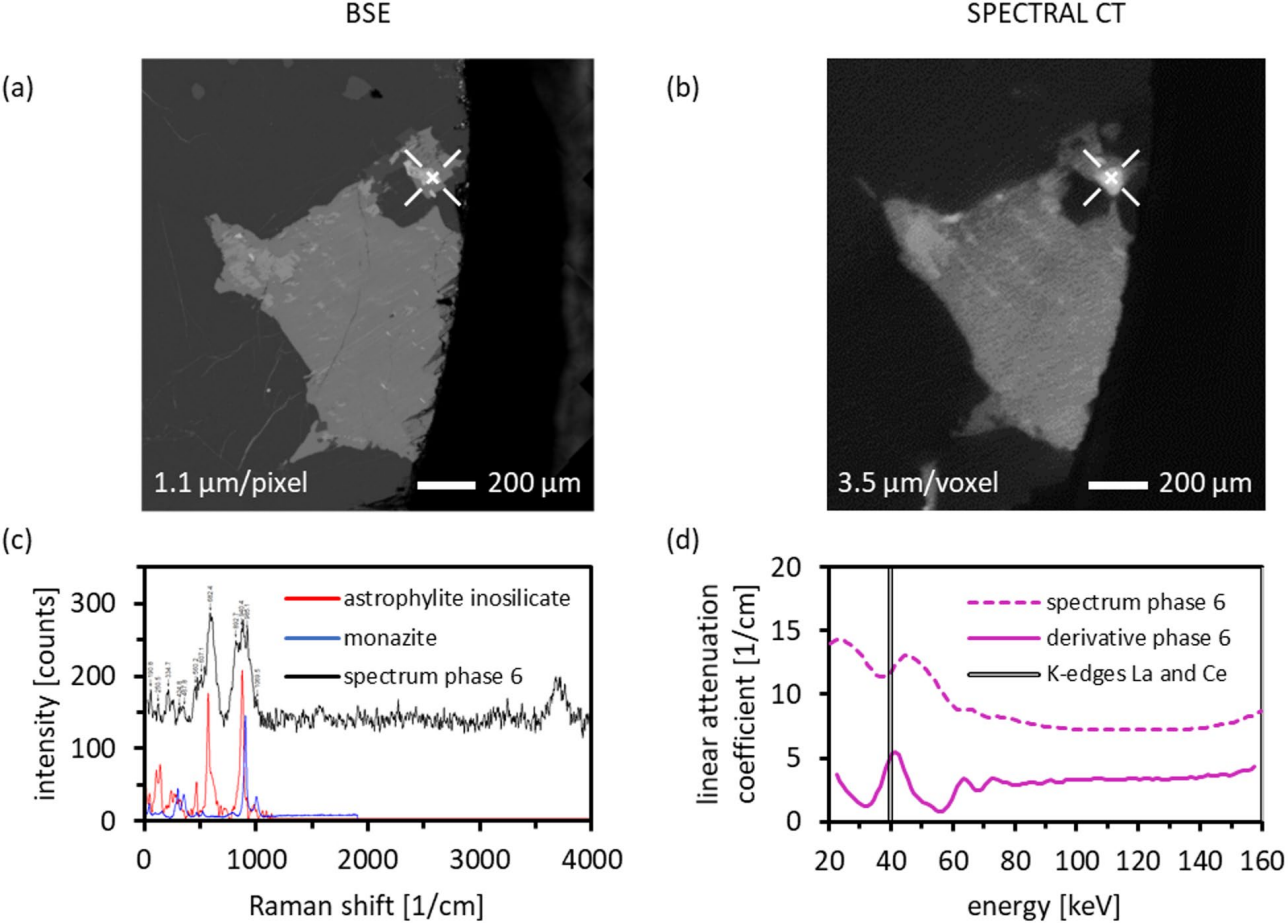
Identification of monazite and astrophyllite using sp-CT: (**a**) SEM BSE image of the zone containing monazite and astrophyllite mixture and (**b**) spectral CT image at 50 keV; (**c**) µRaman spectra showing the characteristic peaks of monazite and astrophyllite; (**d**) energy-dependent linear attenuation coefficient and its derivative showing a characteristic peak corresponding to the K-edge energy of lanthanum (La, Z = 57, K-edge = 38.939 keV)^34^ or cerium (Ce, Z = 58, K-edge = 40.446 keV)^34^.

So far, we have shown that sp-CT enables meaningfully segmenting common rock forming minerals. We remind that the presence of characteristic features in their spectrum enables identifying their elemental composition. As an example, we focus on phase 6. Figure 7a and b show a grain containing phase 6, as imaged with BSE and sp-CT, respectively. The derivative of the spectrum obtained by sp-CT, at the position indicated by the cross on Fig. 7b, contains a peak at 39 keV which could correspond to lanthanum (La, Z = 57, K-edge = 38.939 keV)^34^ or cerium (Ce, Z = 58, K-edge = 40.446 keV)^34^ (Fig. 7d). µRaman spectroscopy at the same location confirmed that this zone contains a mixture of astrophyllite (general formula: (K, Na)_3_(Fe^2+^,Mn)_7_Ti_2_Si_8_O_24_(O, OH)_7_) and monazite (general formula: (Ce, La, Th)PO_4_), thus corroborating the sp-CT observation (Fig. 7c).

## Discussion

We have demonstrated that laboratory sp-CT is a very promising, non-invasive, technique to screen unknown samples for a maximal differentiation between phases. In a first step, one can look at the energy-dependent linear attenuation coefficient obtained in different areas of a sp-CT slice to get an estimation on the number of phases present (Figs. 1d and 2b). In addition, phases containing an absorption edge within the energy range of the screening are revealed (phase 6 in Fig. 2b). In a second step, the multi-energy histogram enhances the differentiation, leading to a precise segmentation of the different phases (Fig. 4c). Absorption edges allow to identify heavy elements. At the time of writing, precise identification of unknown phases without absorption edges still requires coupling with complementary characterisation techniques.

We have illustrated our sp-CT analysis on a monzo-diorite rock, and validated the analysis by means of independent chemical and mineralogical characterization using SEM EDS and µRaman. Conventional micro-CT allowed to threshold four distinct phases, based on their grey tone differences, as shown in the histogram in Fig. 1b. These phases can be attributed to the major minerals found in the monzo-diorite rock, being (1) feldspar, (2) pyroxene and amphibole, (3) fayalite, and (4) Fe-oxides. Sp-CT and segmentation of the multi-energy histogram refined the differentiation, allowing:

- to distinguish between two phases associated to different feldspars, namely plagioclase (phase 1) and orthoclase (phase 2): despite the almost identical spectra over the full energy range of the spectral analysis (Fig. 2b, green and blue curve), the multi-energy histogram (Fig. 4a) permits a clear segmentation of those two minerals, which exhibit the lowest attenuation in the monzo-diorite;
- to distinguish between phases 3, 4 and 9, respectively associated to pyroxene, amphibole and pyroxmangite. These phases have an attenuation in between the feldspars and fayalite, and show slightly differing spectra (Fig. 2b, purple, yellow and red curve);
- to reveal the presence of two distinct phases in areas previously identified as Fe-oxides that could be associated to astrophyllite + monazite (phase 6) and phlogopite (phase 7): these phases show clearly distinct spectra (dashed curves in Fig. 2b), with phlogopite showing the highest attenuation of all measured spectra, and astrophyllite + monazite associated with the only spectrum that contains an absorption edge.

Our results show that the sp-CT technique can be used successfully to differentiate phases that cannot be accurately segmented using conventional micro-CT. For the specific case of the monzo-diorite rock, we can segment nine phases instead of the four found with conventional micro-CT. In specific cases, sp-CT furthermore allows for material identification through the detection of characteristic absorption edges, combined with prior knowledge on the expected sample composition. This was the case for astrophyllite + monazite (phase 6) in our work, and for minerals such as barite, galena and others in the work of Godinho et al.^37^, Sittner et al.^35^ and Buyse et al.^36^. The minimum measurable energy with the sp-CT imaging setup used in our study is 20 keV, which is equivalent to the K-edge of molybdenum (Mo, Z = 42, K-edge = 20.0088 keV)^34^. By differentiating common rock forming silicate minerals that are composed of elements lighter than molybdenum, independent of their K-edge energies, our work goes a step further than the current state of the art. To transition from differentiation to identification, either prior knowledge on the composition of the material of interest is currently required, or complementary analyses need to be performed.

It could be argued that conventional micro-CT scans employing a low source voltage would also enable the discrimination and segmentation of light-element-bearing materials. However, heavy-element-bearing minerals such as astrophyllite, phlogopite or iron-oxide would not feature contrast on such scans. The main advantage of sp-CT in general and the proposed methodology in particular is that a wide range of minerals can be discriminated and that the high-Z elements they are bearing can be identified.

The spectral analysis performed in our study was done on one 2D cross section of a 3D sample. Statistics and discriminating potential could be enhanced further by acquiring multiple cross sections. Note that two-dimensional spectral detectors already exist, enabling the acquisition of a 3D dataset in a single scan, albeit with a lower number of energy bins per voxel (typically 2 or 3, to be compared to 140 in our case). As the technology matures, this limitation can be expected to be lifted.

Another limitation of our work lies in the relatively coarse spatial resolution achieved (15 μm/voxel) for a 8.75 mm diameter sample. Future generations of spectral detectors can be expected to feature a larger number of pixels with smaller physical dimensions. The resulting spectral datasets would feature a higher spatial resolution for the same sample size. As the obtained spectra are inherently volume-averaged over each voxel, smaller voxel sizes therefore enable detecting finer mineral textures and render the resulting multi-energy histogram less prone to partial volume effects.

Regardless the limitations, the proposed methodology opens up to a plethora of new possibilities, as it paves the way for three-dimensional, non-invasive material characterisation. It presents itself as a powerful alternative to conventional dual energy scanning using laboratory^18–21^ or synchrotron-based X-rays^22^, and is less time consuming than correlative imaging, where chemical information from 2D chemical maps is propagated into 3D micro-CT volumes^12,27–29,45^.

Spectral CT is most advantageous (i) to detect heavy-element-bearing phases (typically white spots on conventional micro-CT) based on their absorption edge, and (ii) to separate between phases having combinations of composition and (apparent) density that yield the same overall attenuation. The former has applications in the analysis of ores and the latter in the study of carbonates, for example. The case study of the monzo-diorite illustrates the potential of the sp-CT technique for the field of earth sciences. About 95% of the Earth’s crust is composed of igneous rocks or their metamorphic equivalents that are made up of quartz, feldspar, mica, olivine, pyroxene, and amphibole that are considered as the common rock forming minerals. Those minerals are known to have similar X-ray attenuation coefficients, hardly distinguishable using conventional micro-CT. The method proposed here unlocks the barrier to study such a vast variety of rocks, their minerals, structures and reactions in three dimensions, non-invasively, while allowing to obtain quantitative information. Therefore, the proposed methodology is of primary interest to petrologists and mineralogists, for example, to characterize meteorites, or for 3D or 4D studies of fluid-rock interactions applied to mineral carbonation^43,46^ or natural hydrogen exploration.

## Methods

### Monzo-diorite sample

We characterized a fayalite-bearing monzo-diorite obtained from the Precambrian basement of Kansas, USA. Petrographic microscopy and SEM-EDS analysis^44^ revealed that this rock consists of orthoclase and plagioclase feldspar with assemblages of fayalite, pyroxene, amphibole and metal oxides as the major mineral phases. Pyroxene and amphibole are mostly clustered around fayalite throughout the rock. The rock is cut by veins of Fe-Si rich minerals, identified as a mixture of serpentine+chlorite+micas and interstratified layers of chlorite+smectite. A small cylindrical sample with a diameter of 8.75 mm and height of 17.00 mm was randomly drilled. A horizontal plane containing numerous minerals of interest was selected by 3D visualizing the entire volume with X-ray microtomography imaging. Then, the sample was cut and polished to expose the selected plane. More information on the sample, its preparation and first characterisation can be found in^30^.

### Conventional and spectral computed tomography

Conventional micro-CT and spectral CT (sp-CT) were performed on the monzo-diorite sample using a Tescan UniTOM XL Spectral (UPPA, DMEX Center for X-ray Imaging, Pau, France). This instrument is equipped with a polychromatic X-ray source and two detectors, a 16-bit flat panel detector of 2856 × 2856 pixels and a linear CdTe-detector featuring a sensor width of 307 mm, yielding projection images with 764 × 1 pixels and 140 channels. Conventional micro-CT imaging was performed at 60 kV tube voltage and a target power of 14 W, using an exposure time of 380 ms and targeting a voxel size of 3.5 μm. A series of 4282 angular projections were collected over a 360° sample rotation. Each angular projection is the average of 2 projection images, resulting in a total scanning time of 58 min. The acquired data were reconstructed using Panthera (v 1.4.3.16, Tescan). A spectral tomography slice was acquired at the same position as the visualized slice of the conventional micro-CT dataset (slice shown in Fig. 1), just below the polished surface, operating the X-ray source at 160 kV and a target power of 20 W. A series of 1201 angular projections were collected over a 360° sample rotation. The total acquisition time was 63 min and the reconstructed voxel size 15 μm/voxel. The reconstructed linear attenuation coefficient at each voxel is comprised of 140 equidistant energy bins of 1 keV between 20 and 160 keV. The voxel size of the spectral scan was selected in order to cover the full cross-section of the sample, as in the conventional scan, hereby sacrificing the detection of the finer features of the microstructure.

### SEM analysis

Scanning electron microscopy (SEM) analysis was performed to obtain a back-scattered electron (BSE) image and the energy dispersive X-ray spectroscopy (SEM EDS) element distribution maps of Na, Mg, K and Fe of the exposed surface of the monzo-diorite sample. A MEBFEG JEOL JSM 7100 F equipped with a field emission gun (FEG) was used at the Raimond Castaing Microanalysis Centre, Toulouse, France. The total surface area mapped was approximately 60 mm^2^. The resolution of each map was approximately 1.18 μm.

### Micro Raman analysis

Micro Raman (µRaman) spectroscopy was performed on the polished surface of the natural monzo-diorite sample using a Horiba Scientific Xplora Raman spectrometer (Institute des Sciences Moléculaires, University of Bordeaux, France) in order to identify the mineralogical composition of the different phases. Point analysis was performed using a 532 nm laser beam, with a grating of 1800 lines/mm, a confocal hole aperture of 280 μm and a slit of 100 μm. The objective was at a magnification of x50, resulting in a spatial resolution of about 10 μm and a spectral resolution of 5 μm. The acquisition time amounted to 60 s with a laser power of 10 mW. The data were processed using LabSpec 6 (Horiba Scientific) software to remove fluorescence-related background noise. Subsequent peak search-matching was performed using Spectral ID (version 3.0, Galactic Industries Corporation). Locations of µRaman point analysis marked on the phases segmented using the sp-CT method are given in Fig. 5 together with the minerals identified with the spectral searching.

µRaman mapping at the contact between pyroxene and amphibole (Fig. 6) was performed using a LabRam Image Raman micro spectrometer from Horiba Jobin-Yvon, analysis wavelength 514 nm, grating 1800 lines/mm, confocal hole aperture 280 μm and slit 100 μm, objective magnification x50, giving a spatial resolution of approximately 10 μm and a spectral resolution of 5 μm, with an acquisition time of 60 s and laser power 10 mW. Raman spectra were recorded in a 40 × 8 μm area with a 4 μm step. Then, a Raman band of the compound of interest was selected and its relative intensity was plotted along this analysis area in a coloured graph. The more the colour is red, the more the intensity of the Raman band is important, and conversely, the more the colour is blue-black, the less the intensity of this Raman band is. We thus obtain a vibrational image which corresponds to the distribution of the compound in the analysed area.

## Data Availability

The spectral dataset generated and analysed during the current study is publicly available on https://doi.org/10.57745/CX3ELY.

## Code availability

The in-house developed software for the spectral data analysis is publicly available on the Git repository of the Université de Pau et des Pays de l’Adour at https://git.univ-pau.fr/dmex/segmentSpectralData/.
